# Intermittent hypoxic training improves anaerobic performance in competitive swimmers when implemented into a direct competition mesocycle

**DOI:** 10.1371/journal.pone.0180380

**Published:** 2017-08-01

**Authors:** Miłosz Czuba, Robert Wilk, Jakub Karpiński, Małgorzata Chalimoniuk, Adam Zajac, Józef Langfort

**Affiliations:** 1 Department of Sports Training, the Jerzy Kukuczka Academy of Physical Education in Katowice, Faculty of Physical Education, Katowice, Poland; 2 Department of Swimming, the Jerzy Kukuczka Academy of Physical Education in Katowice, Faculty of Physical Education, Katowice, Poland; 3 Department of Tourism and Health in Biala Podlaska, Józef Piłsudski University of Physical Education in Warsaw, Warsaw, Poland; University of Kentucky Medical Center, UNITED STATES

## Abstract

The main objective of this research was to evaluate the efficacy of intermittent hypoxic training (IHT) on anaerobic and aerobic capacity and swimming performance in well-trained swimmers. Sixteen male swimmers were randomly divided into a hypoxia (H) group (n = 8), which trained in a normobaric hypoxia environment, and a control (C) group (n = 8), which exercised under normoxic conditions. However, one participant left the study without explanation. During the experiment group H trained on land twice per week in simulated hypoxia (FiO_2_ = 15.5%, corresponding to 2,500 m a.s.l); however, they conducted swim training in normoxic conditions. Group C performed the same training program under normoxic conditions. The training program included four weekly microcyles, followed by three days of recovery. During practice sessions on land, the swimmers performed 30 second sprints on an arm-ergometer, alternating with two minute high intensity intervals on a lower limb cycle ergometer. The results showed that the training on land caused a significant (p<0.05) increase in absolute maximal workload (WR_max_) by 7.4% in group H and by 3.2% in group C and relative values of VO_2max_ by 6.9% in group H and 3.7% in group C. However, absolute values of VO_2max_ were not significantly changed. Additionally, a significant (p<0.05) increase in mean power (P_mean_) during the first (11.7%) and second (11.9%) Wingate tests was only observed in group H. The delta values of lactate concentration (ΔLA) after both Wingate tests were significantly (p<0.05) higher in comparison to baseline levels by 28.8% in group H. Opposite changes were observed in delta values of blood pH (ΔpH) after both Wingate tests in group H, with a significant decrease in values of ΔpH by 33.3%. The IHT caused a significant (p<0.05) improvement in 100m and 200m swimming performance, by 2.1% and 1.8%, respectively in group H. Training in normoxia (group C), resulted in a significant (p<0.05) improvement of swimming performance at 100m and 200m, by 1.1% and 0.8%, respectively. In conclusion, the most important finding of this study includes a significant improvement in anaerobic capacity and swimming performance after high-intensity IHT. However, this training protocol had no effect on absolute values of VO_2max_ and hematological variables.

## Introduction

Over the past few years, intermittent hypoxic training (IHT) has been recognized as an effective method to improve performance in sport disciplines that require a high level of aerobic and/or anaerobic endurance. In IHT, athletes train or are exposed to simulated normobaric hypoxia or less often in a natural high-altitude environment under hypobaric conditions, while living under normoxic conditions [1]. Compared to other well-known methods of altitude training IHT presents a few essential advantages that can be utilized as an integral component of modern athletic training, aimed at peak performance. Among them the most evident are: 1) IHT prevents athletes from sleeping disorders and dehydration, which are typical symptoms seen during an extended stay at altitude when other models of altitude training are applied [2], 2) recovery following IHT training sessions occurs under normoxic conditions, which prevents athletes from deleterious effects of prolonged hypoxia and shortens the post-training recovery time, and 3) the time spent apart from training under hypoxic conditions may be used for normal training activity [3,4].

The mechanisms underlying the improvement in athletes’ performance at sea level with altitude training are generally attributed to either cardiovascular [5], hematological [6], or ventilatory [7] effects. A strong emphasis on elevation of erythrocyte mass and hemoglobin concentration is considered as a pivotal and predominate factor for improvement of oxygen transport in the body and thereby VO_2max_ and athletic endurance performance [8]. Despite a vast majority of available data that hypoxic/altitude exposure is erythropoietic, there is still debate on a cause-and-effect relationship between erythropoiesis and enhanced athletic performance. For example, native Ethiopian residents at 3,530m show no meaningful rise in EPO, hemoglobin and oxygen saturation compared to the values of U.S. sea level population, despite their lasting exposure to decreased ambient oxygen tension [9]. Regardless of the exact cause, the remarkable performance capabilities of Ethiopian athletes may be at least partially explained by non-hematological adaptations [10]. Such a phenomenon was observed in well controlled experiments [11,12] including our previous studies [1,3], and was attributed to peripheral adaptations, i.e. muscle buffering capacity [13], glycolytic enzyme activity [14] and mechanical efficiency [15]. It is worth noticing that effective duration of altitude training is close to four weeks for blunted hypoxic pulmonary vasocontraction, increased erythropoiesis and reprogramming for the suppression of ATP turnover rate [16], while three weeks is sufficient for beneficial effects in economy [17], muscle buffering capacity [18], the hypoxic respiratory response [19] and biochemical changes within skeletal muscle [20].

Recent studies showed that exposure to hypoxia during IHT for a short period (1–2 hours) was insufficient to significantly stimulate erythropoiesis [1,3] yet it increased the activity of glycolytic enzymes [1,3,21,22], lactate exchange and removal, as well as tissue O_2_ extraction [16,17,18]. Some data also showed that IHT protocol improved repeated sprint ability (RSA), and rate of phosphocreatine resynthesis [16,23,24]. All aforementioned results suggest that IHT applied in combination with high intensity exercise would constitute a new perspective to potentiate anaerobic performance in athletes. In line with such an assumption is the study showing that repeated sprint training in hypoxia improves repeated agility performance compared to normoxic training [25].

However, there are still many controversies regarding the mechanisms of hematological and nonhematological adaptations to IHT and the extent of enhanced athletic performance. The predominance of studies investigating adaptation to IHT have focused on sport disciplines that comprise the endurance type of exercise as a principal measure of training process, while data concerning the effect of hypoxic training on anaerobic performance are very limited. Therefore, the present study attempted to verify if a three week mesocycle of IHT training would modify hematological and nonhematological variables in sprint swimmers. The mesocycle applied by our athletes was design to stimulate speed, and anaerobic endurance in a direct competition preparation mesocycle (DCPM).

## Materials and methods

### Participants

Sixteen male sprint swimmers with at least six years of training experience participated in this study, but one participant left the study without explanation. All subjects were randomly divided into a hypoxia (H) group (n = 8; age 19.1 ± 1.3 years; VO_2max_ 56.1 ± 4.5 ml/kg/min; body height (BH) 183.5 ± 3.2 cm; body mass (BM) 76.4 ± 5.4 kg; fat content (FAT%) 9.3 ± 3.5%), who trained in a normobaric hypoxia environment, and a control (C) group, which exercised under normoxic conditions (n = 7; age 20.5 ± 1.3 years; VO_2max_ 54.0±6.2 ml/kg/min; BH 181.8 ± 4.2 cm; BM 74.1 ±6.3 kg; FAT% 9.5 ± 1.6%). All athletes had current medical examinations, without any contraindications to performing exhaustive exercise in a hypoxic environment. Volunteers provided their written, voluntary, informed consent before participation.

The research project was conducted according to the Helsinki Declaration and was approved by the Ethics Committee for Scientific Research at the Jerzy Kukuczka Academy of Physical Education in Katowice, Poland.

### Experimental design

The research was conducted during the competitive season. The experiment was divided into two series of tests performed in a laboratory environment and in a 25m swimming pool, separated by five microcycles (31 days). All participants were familiarized with the test protocol one week before the first evaluations. Then, four microcycles (four weeks) with a progressive training load were applied, followed by a short recovery microcycle (three days). The final evaluations were performed after the recovery microcycle. The testing procedures in both series were identical for all participants.

### Testing protocol

This research project used two test series (S1, S2), with a pre-test between four and one days before training (S1), and the second test at three days post-training (S2). Both test series (S1, S2) included three days of examinations in normoxia. Additionally, one day before the training program was initiated (during S1), all athletes in the H group preformed the ramp test in hypoxic conditions (2500m) to establish individual training loads for IHT sessions.

On the first day of each test series (S1, S2), the participants performed a 15 minute warm-up, followed by both a 100m and 200m freestyle swimming test. Electronic timing was used during the test by means of the Omega Electronics OCP5 touch pad. The recovery period between the 100m and 200m tests was 6 hours.

On the second day of evaluations, before breakfast and after an overnight fast, resting blood samples were drawn from the antecubital vein to determine hematological variables, such as hemoglobin concentration (HGB), hematocrit value (HCT), and number of erythrocytes (RBC) (Advida 2120, Siemens, Germany). Next, body mass and body composition were evaluated using the electrical impedance technique (Inbody 720, Biospace Co., Japan). Two hours after a light breakfast (300–350 kcal, 50% carbohydrates, 20% proteins, 30% fats), the ramp cycle ergometer test was performed to determine aerobic capacity.

The ramp test was performed on an Excalibur Sport cycle ergometer (Lode, Netherlands), starting from a workload of 50 W, which was increased linearly by 25 W per minute (0.42 W/s) until volitional exhaustion. During the test, heart rate (HR), oxygen uptake (VO_2_), expired carbon dioxide (CO_2_) and minute ventilation (VE) were measured continuously using the MetaMax 3B telemetry spiroergometer (Cortex, Germany) in the breath-by-breath mode. The criteria of reaching VO_2max_ included a plateau in the level of VO_2_ or a gradual decrease in peak VO_2_ during the maximal workload.

Fingertip capillary blood samples for the assessment of lactate (LA) concentration (Biosen C-line Clinic, EKF-diagnostic GmbH, Germany) were drawn at rest and at the end of each test, as well as during the 3^rd^, 6^th^, 9^th^, and 12^th^ min of the recovery. Additionally, capillary rest and post-exercise blood samples were used to determine acid-base equilibrium and oxygen saturation of hemoglobin (RapidLab 248, Bayer Diagnostics, Germany).

On the third day of S1 and S2, a double 30 second Wingate test for upper limbs was performed to determine anaerobic capacity. The tests, preceded by a five minute warm up (50W), were performed on an electromagnetically braked ergometer (Brachumera Sport, Lode). The Wingate tests were performed with the resistance adjusted to the athlete’s body weight (0.45Nm/kg). Five minutes of active recovery (50W) was applied between the first and the second Wingate test. Capillary blood samples were drawn at rest and three minutes after the second Wingate test, to determine changes in lactate concentration, acid-base equilibrium and oxygen saturation of hemoglobin. Capillary blood samples were also drawn after 6^th^, 9^th^ and 12^th^ minute following the second Wingate test to determine lactate utilization.

After 24 hours of rest in S1, athletes in the experimental group (H) performed the same ramp test protocol as on the second day, but in a normobaric hypoxia chamber at 2,500m (FIO_2_ = 15.5%) to establish individual training loads for IHT sessions (%VO_2max_hyp).

The ambient conditions concerning the temperature (18.9°C–S1; 19.1°C- S2) and humidity (51%—S1; 52%- S2) were maintained at a constant level in both test series (S1, S2) to increase measurement reliability.

### Training program

The training program included four microcycles (four weeks) with progressive training loads, followed by a short recovery microcycle (three days). Intermittent hypoxic training (IHT) with high intensity took place twice per week, where the prevalence of O_2_ was 15.5%, corresponding to 2,500m a.s.l. The C group performed the same training program under normoxic conditions. Training intensity was selected individually, based on the results of initial aerobic capacity tests. Each training session consisted of a 10 minute general warm-up, 45–55 minute main part, and a 10 minute cool-down. The main part of the circuit consisted of exercise performed on an upper limb rotator (50 W) with a cadence of 80 rpm lasting 60 seconds. Then the swimmers performed a 30 second maximum effort on the rotator from a flying start with a load of 0.4 Nm·kg^-1^ (Brachumera Sport, Lode). After a 30 second rest period, the athletes performed a three minute ride on a cycle ergometer (Cyclus 2) with the intensity set at 50% VO_2max_hyp/VO_2max_. Following a three minute period of active rest on the cycle ergometer, the swimmers performed another two minute exercise bout, at an intensity of 95% VO_2max_hyp/VO_2max_. After the high intensity phase of training for the lower limbs, the athletes peddled for three minutes at the intensity of 50% VO_2max_hyp/VO_2max_. This circuit was repeated four times in the first four interval training sessions, after which a 5^th^ circuit was added to increase the overall training load. The swimming training was the same for athletes in both the normoxic and hypoxic groups with a volume of approximately 50 km per week (Table 1).

**Table 1:** Training program during the experiment.

Day	Microcyle 1	Microcyle 2	Microcyle 3	Microcyle 4
1.	AM: 1h –TL4 + 30min swimming REC PM: 2h –swimming EN2	AM: 1h –TL4 + 30min swimming REC PM: 2h –swimming EN2	AM: 1:15h –TL5 + 45min swimming REC PM: 2h –swimming EN2	AM: 1:15h –TL5 + 45min swimming REC PM: 2h –swimming EN2
2.	AM: 1h –ST + 1h EN3 swimming PM: 2h –swimming EN2	AM: 1h –ST + 1h EN3 swimming PM: 2h –swimming EN2	AM: 1h –ST + 1h EN3 swimming PM: 2h –swimming EN2	AM: 1h –ST + 1h EN3 swimming PM: 2h –swimming EN2
3.	AM: 2h –SP1 swimming PM: off	AM: 2h –SP1 swimming PM: off	AM: 2h –SP1 swimming PM: off	AM: 2h –SP1 swimming PM: off
4.	AM: 1h –TL4 + 30min swimming REC PM: 2h –swimming EN2	AM: 1h –TL4 + 30min swimming REC PM: 2h –swimming EN2	AM: 1:15h –TL5 + 45min swimming REC PM: 2h –swimming EN2	AM: 1:15h –TL5 + 45min swimming REC PM: 2h –swimming EN2
5.	AM: 1h –ST + 1h EN3 swimming PM: 2h –swimming EN2	AM: 1h –ST + 1h EN3 swimming PM: 2h –swimming EN2	AM: 1h –ST + 1h EN3 swimming PM: 2h –swimming EN2	AM: 1h –ST + 1h EN3 swimming PM: 2h –swimming EN2
6.	AM: 2h –SP1 swimming PM: off	AM: 2h –SP1 swimming PM: off	AM: 2h –SP1 swimming PM: off	AM: 2h –SP1 swimming PM: off
7.	ff	off	off	off

TL4 –Training in the lab—4 circuits, TL5 –Training in the lab—5 circuits, ST–core stability training, REC–recovery training (up to 75% HR_LT_), EN2 –endurance training (75–85% HR _LT_), EN3 –endurance training (95–105% HR _LT_), SP1 –anaerobic capacity training, SP3 –speed training.

### Statistical methods

The results of the study were analyzed by means of StatSoft Statistica 12.0 software. The results were presented as arithmetic means (x) with standard deviations (SD). Statistical significance was set at p<0.05. The Lilliefors test was used to demonstrate the consistency of the results obtained in the study with normal distribution. The intergroup differences between research series were determined using the multi-factor analysis of variance (MANOVA) for repeated measures. Significance of differences between individual research series in the study groups was calculated based on the post-hoc Tukey's test.

## Results and discussion

### Results of ANOVA

A two-way analysis of variance in the group × training interaction showed significant differences in the absolute maximal workload (WR_max_; F = 9.671, p = 0.008), relative values of maximal oxygen uptake (VO_2max_; F = 6.340, p = 0.025), as well as in blood lactate concentration (ΔLA; F = 20.183, p = 0.001) and blood pH changes (ΔpH; F = 4.865, p = 0.046) observed during the ramp test.

**Fig 1:**
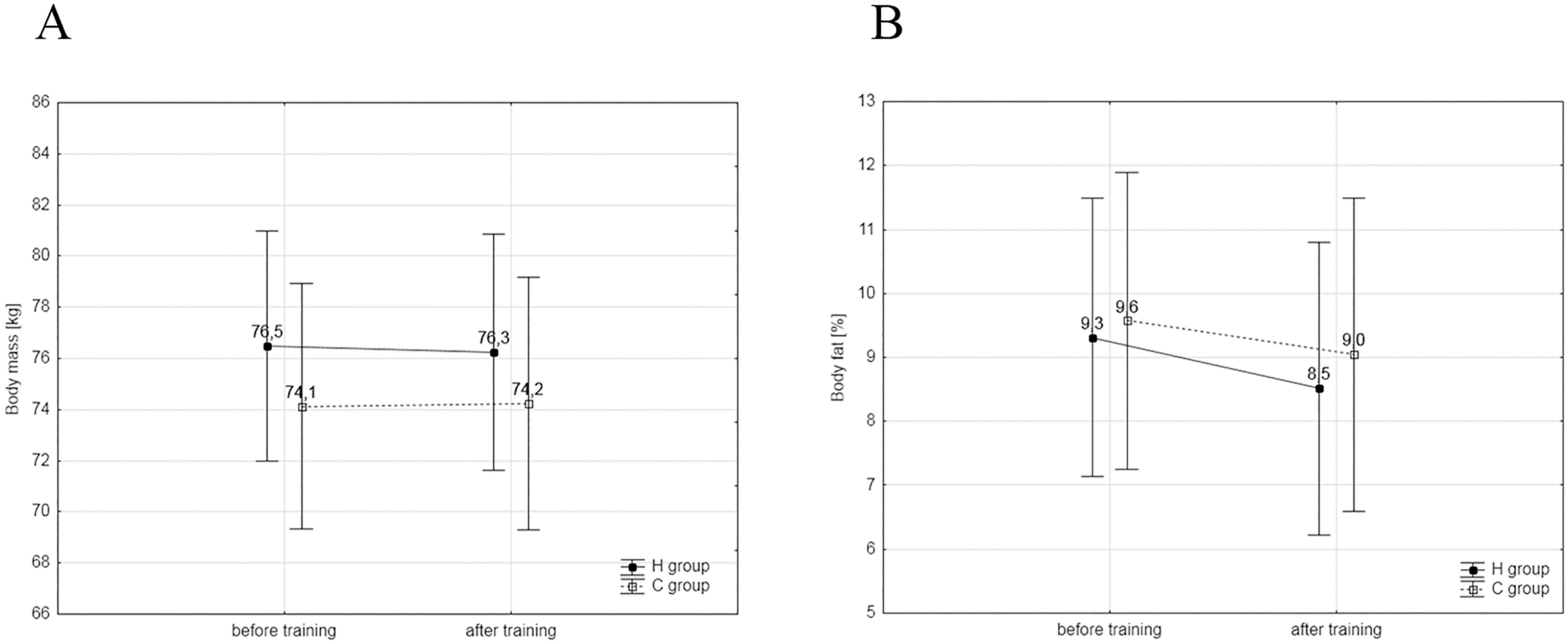
A, B. Body mass and composition. (A)Values of body mass and in the experimental (H, n = 8) and control groups (C, n = 7). (B)Values of body fat in the H and C groups.

Other significant differences (group × training interaction) were found in absolute mean power of the first (F = 5.634 p = 0.0299) and second Wingate tests (F = 12.109 p = 0.004), as well as significant differences in the increase in blood lactate concentration (ΔLA, F = 7.073; p = 0.0196), in blood pH changes (ΔpH, F = 8.270; p = 0.0129). The results of the 100m (F = 29.7; p = 0.0001) and 200m (F = 87.94; p = 0.0001) swim tests were also significantly different. The training plan did not have a significant effect on changes in body mass and body composition (Fig 1A and 1B) as well as in selected hematological variables (Table 2).

**Table 2:** Mean values of selected hematological variables in the experimental (H, n = 8) and control groups (C, n = 7).

Variable	Group H		Group C	
	Before training (S1)	After training (S2)	Before training (S1)	After training (S2)
RBC (mln/μl)	5.05 ± 0.19	5.11 ± 0.27	5.27 ± 0.31	5.25 ± 0.31
HGB (g/dl)	15.4 ± 0.4	15.6 ± 0.6	15.6 ± 0.8	15.5 ± 0.9
HCT (%)	45.9 ± 4.2	46.2 ± 2.1	46.3 ± 2.4	46.1 ± 1.8

RBC–red blood cell count, HGB- hemoglobin, HCT–hematocrit

### Post-hoc analysis

The *post-hoc* analysis showed that IHT caused a significant (p<0.001) increase in absolute maximal workload (WR_max_) by 7.4% in group H and by 3.2% in group C (p<0.05). Also, the relative values of VO_2max_ in both groups increased significantly (p<0.05) by 6.9% in group H and 3.7% in group C. The delta values in ΔLA after the ramp test were significantly (p<0.05) higher in both groups, in comparison to baseline levels by 20.9% in group H and by 7.2% in group C. Additionally, the statistical analysis also revealed a significantly (p<0.01) higher decrease in blood pH values after the ramp test in group H by 21.1%. In group C, similar changes in these variables were not found (Table 3).

**Table 3:** Changes in maximal workload and selected cardiorespiratory indices recorded in the experimental and control groups (H, n = 8; C, n = 7) during the ramp test; * p<0.05; ** p<0.01; *** p<0.001 –statistically significant differences in relation to initial measurements.

Variable	Group H		Group C	
	Before training (S1)	After training (S2)	Before training (S1)	After training (S2)
WR_max_ (W)	362.0 ± 22.2	388.7*** ± 18.5	352.8 ± 35.6	364.0* ± 37.2
VO_2max_ (l/min)	4.28 ± 0.29	4.54 ± 0.31	3.97 ± 0.41	4.14 ± 0.49
VO_2max_ (ml/kg/min)	56.0 ± 4.0	59.9*** ± 4.3	54.0 ± 6.2	56* ± 6.7
RER_max_	1.11 ± 0.02	1.12 ± 0.03	1.14 ± 0.04	1.13 ± 0.03
VE_max_ (l/min)	172.7 ± 22.4	187,1 ± 19.1	161.6 ± 19.1	165.8 ± 29.4
HR_max_ (bpm)	187 ± 6	192 ± 6	182 ± 10	183 ± 9
ΔLA (mmol/l)	9.14 ± 0.92	11.05*** ± 1.33	9.34 ± 1.26	10.01*± 1.19
ΔLA12’res(mmol/l)	2.91 ± 0.87	3.24 ± 1.08	2.46 ± 0.6	2.98 ± 1.16
ΔpH	-0.147 ±0.051	-0.178** ±0.048	-0.149 ±0.041	-0.153 ± 0.057
O_2_Sat (%)	95.7 ±1.0	94.7 ±1.3	95.3 ± 0.9	94.9 ± 1.3

WR_max_—maximal workload during ramp test, VO_2max_—maximal oxygen uptake, RER_max_−maximal respiratory ratio during ramp test, VE_max_—maximal ventilation, HR_max−_maximal heart rate, ΔLA—increase in blood lactate concentration during ramp test, ΔLA12’res–decrease in blood lactate concentration after 12’ of recovery, ΔpH—blood pH changes, O_2_Sat- oxygen saturation at the end of the ramp test.

**Fig 2:**
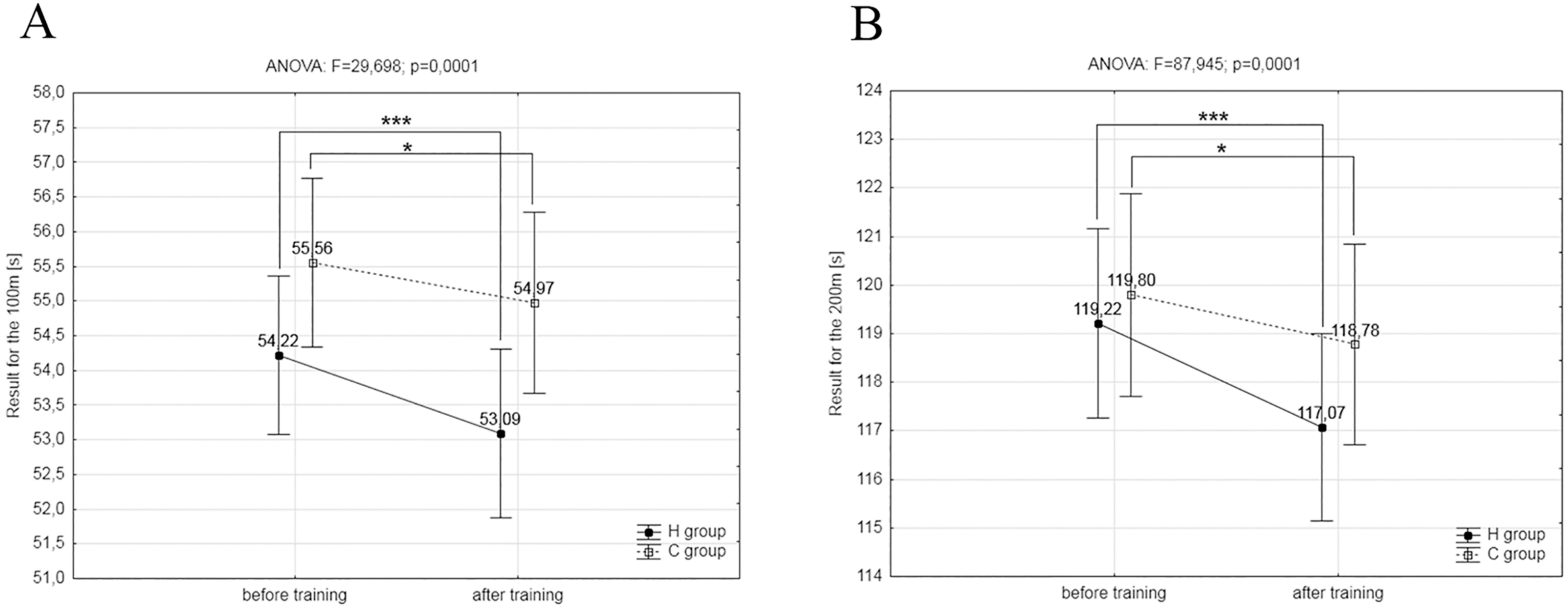
A, B. The results of the swimming tests. (A) The results of the 100m swimming tests in the experimental (H, n = 8) and control groups (C, n = 7) before and after the experiment. (B) The results of the 200m swimming tests in the H and C groups before and after the experiment; *-p<0.05, *** p<0.001—significant differences in relation to initial measurements.

Furthermore, the *post-hoc* Tukey’s test showed a significant increase (p<0.001) in mean power (P_mean_) during the first (11.7%) and second (11.9%) Wingate tests in the group H. The delta values in lactate concentration (ΔLA) after both Wingate tests were significantly (p<0.05) higher in comparison to baseline levels by 28.8% in the group H. Opposing changes were observed in delta values of blood pH (ΔpH) after both Wingate test in group H, with a significant (p<0.01) decrease in values of ΔpH by 33.3%. In group C, similar changes in these variables were not found (Table 4). The *post-hoc* analysis showed that the IHT caused a significant (p<0.001) improvement in 100m and 200m swimming performance, by 2.1% and 1.8%, respectively (Fig 2A and 2B). Similar but significant minor changes were also observed after training in normoxia, causing a significant (p<0.05) improvement in swimming performance at 100m and 200m, by 1.1% and 0.8%, respectively (Fig 2A and 2B).

**Table 4:** Results of the double Wingate test and selected biochemical variables in the experimental and control groups (H, n = 8; C, n = 7); * p<0.05; ** p<0.01; *** p<0.001—statistically significant differences in relation to initial measurements.

Variable	Test	Group H		Group C	
		Before training (S1)	After training (S2)	Before training (S1)	After training (S2)
P_peak_ (W)	I	741.0 ± 124.3	809.3 ± 103.9	762.2 ± 146.2	887.6 ± 162.9
	II	657.3 ± 82.6	793.1 ± 109	670.5 ± 143.1	818.2 ± 208.2
P_mean_ (W)	I	452.7 ±37.6	505.8*** ±48.3	433.0 ± 37.7	454.1 ± 35.4
	II	425.9 ±25.9	477.0*** ±32.3	398.2 ± 25.0	413.3 ± 24.2
ΔLA (mmol/l)	3’ after II	9.16 ± 2.44	11.8* ± 2.10	12.67 ± 2.48	11.80 ± 1.74
ΔLA12’res(mmol/l)	II	2.20 ±0.67	1.96 ±0.60	2.10 ± 0.97	2.15 ± 0.78
ΔpH	3’ after II	-0.157 ±0.032	-0.223** ± 0.054	-0.189 ± 0.051	-0.201 ± 0.047
O_2_Sat (%)	3’ after II	95.9 ±0.2	96.1 ±0.2	94.6 ± 3.3	95.1 ± 3.5

P_peak_−peak power, P_mean_−mean power, ΔLA—increase in blood lactate concentration after double Wingate test, ΔLA12’res–decrease in blood lactate concentration after 12’ of recovery, ΔpH—blood pH changes after double Wingate test, O2Sat- oxygen saturation after two Wingate tests.

Periodization of the training process is a treatment applied by athletes to optimize training loads and reach peak performance for the main competition [26]. In recent years the vast majority of research investigating the application of altitude/hypoxic training was focused on aerobic endurance outcomes [3,6]. In the present study, we extended this exploration by investigating anaerobic performance in a direct competition preparation mesocycle (DCPM) appended in combination with IHT protocol in competitive swimmers. The main finding of our study is that the 4-week IHT protocol improved anaerobic capacity and swimming performance in a group of competitive athletes measured by means of “field test” (100m and 200m swim trials) and 30 second all-out Wingate test. The study demonstrates significant increases in both absolute and relative MP by ~12% during the first and ~10%, in the second Wingate test, respectively. The above mentioned data and a significant improvement (~2%) in the 100m and 200m freestyle swimming performance suggests a substantial contribution of anaerobic glycolysis to energy supply for the working muscles in both tests. In line with such an assumption is the fact that VO_2max_ and hematological variables were not affected by IHT, thus an improvement of anaerobic exercise capacity could not pertain an elevation of aerobic energy supply during both high intensity exercise tests used in our study. The later possibility may be pondered since Yamamoto and Kanehisa [27] showed that oxygen uptake between 15–30 seconds of a Wingate test reached about 75% of VO_2max_ which clearly indicated that aerobic energy supply is also an important source of muscle energy during high intensity exercise. A less significant response to the swimming performance compared to the Wingate test is likely to be caused by the specific character of the IHT training which was performed outside the swimming pool, what resulted in less pronounced improvements in swimming performance. Therefore, it seems advisable that the IHT training for swimmers should be performed in the water environment using specific training means.

### Anaerobic effects

The efficacy of IHT for the enhancement of sea-level anaerobic performance has yet to be fully investigated. One of the first reports to explore this area was the study conducted by Hamlin et al. [28], who found an increase in mean anaerobic power during the 30 second Wingate test following the IHT procedure of well-trained athletes. Furthermore, the reports published in recent years have demonstrated the effectiveness of the IHT training in terms of repeated sprint ability (RSA) [16,24]. Results obtained in our investigation by means of the Wingate test confirm the aforementioned data, showing that IHT improves anaerobic performance of land-based exercises that were performed in an upright positon with a predominant involvement of lower body muscles in exercise execution. However, the present study extends these findings to whole-body exercise performed in a prone position during freestyle swimming, where the arms provide 70–85% of the propulsive forces [29,30]. Moreover, swimming has been shown to be more energy demanding per unit of distance than locomotion on land and both internal and external work in water is elevated compared to land-based conditions, because of water density [31].

**Fig 3:**
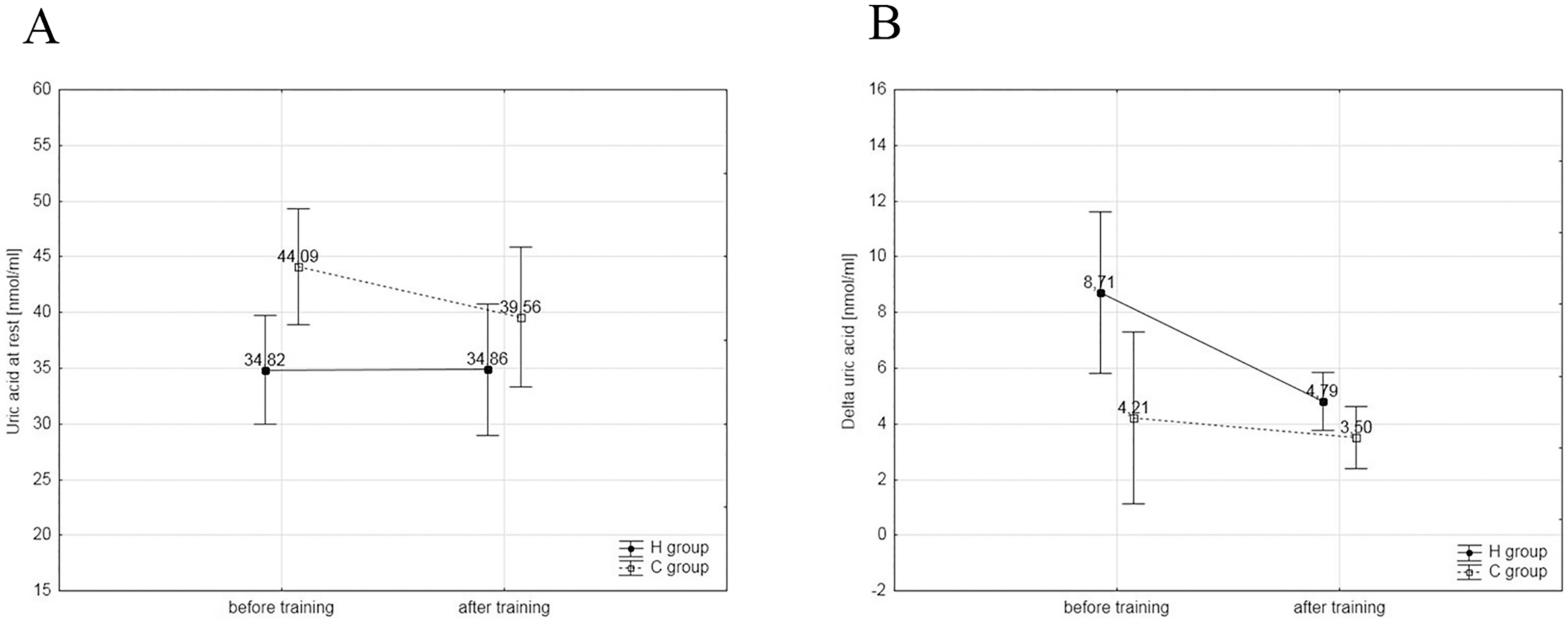
A, B. Changes in uric acid concentration. (A) Changes in resting concentration of uric acid in the experimental (H, n = 8) and control groups (C, n = 7 during the first and last training session. (B) Changes in delta values of uric acid in the H and C groups during the first and last training session.

Albeit the mechanisms for beneficial effects of IHT on anaerobic performance is not fully recognized, some data allow to assume that the enhancement of nonhematological physiological adaptations, with changes at the level of muscle energy metabolism can play a pivotal role [32]. However, a recent study with the use of ^31^P-magnetic resonance spectroscopy has revealed no alteration in PCr concentration between work matched height-intensity, incremental exercise performed in normoxic or hypoxic conditions [33]. This finding agrees with our results that showed no difference in plasma content of uric acid (Fig 3A and 3B), a product of purine nucleotides degradation. It means that a high exercise intensity applied in our study did not trigger activation of adenylate kinase and AMP signaling pathways in both environmental conditions [34]. These findings suggest that intermittent hypoxia-induced challenge to muscle energy homeostasis is compensated by other adaptations to maintain cellular energetics. It is known that the exposure to hypoxia leads to stimulation of HIF-1, which, apart from regulation of erythropoiesis, is also a regulator of activity of glycolytic enzymes, mainly phosphofructokinase (PFK-1) [35], and therefore improvements in anaerobic capacity of muscles may occur. This was confirmed by a study of Meeuwsen et al. [36] and Hendriksen and Meeuwsen [37], who documented that IHT (2 hours/day for 10 days, 2,500m above sea level) led to significant improvements in power generated during a 30 second Wingate test. Similar changes were not demonstrated in the control group that trained under normoxic conditions. Vogt et al. [22] and Zoll et al. [12] also found that six-week IHT training induced not only elevated expression of messenger RNA (mRNA) that codes PFK-1, but also the transporters of glucose 1 (GLUT-1) and regulation of pH (monocarboxylate transporter 1, carbonic anhydrase). However, there are also reports inconsistent with the above observations. This concerns early studies on the IHT protocol [38,39], which were based on less intensive training loads. A decline in blood lactate concentration was found during the exercise, with a decline in PFK-1 activity and lactate dehydrogenase (LDH).

Recent examinations [40] of chronic passive exposure to hypoxia (live high, train low method) suggest that exercise intensity may have a key effect on adaptations in terms of MCT1 and MCT4. The study measured MCT1 and MCT4 protein concentration in highly-trained athletes exposed to hypoxia for 20 nights. Although an increase in the rate of LA utilization was observed, no changes were found in MCT1 and MCT4 concentration. These changes may be attributable to stronger activation of the lactate/H^+^ transport system during exercise compared to rest [41]. Consequently, sleeping in hypoxic conditions is an insufficient stimuli for the increase in MCT1 and MCT4 protein expression. Furthermore, Zoll et al. [12] demonstrated an increase of 44% in the level of mRNA for MCT1 in skeletal muscle of trained runners after the IHT protocol with a high training load (3,000m above sea level, two times a week from 24 to 40 min). No changes were found in the control group that trained under normoxic conditions. Running time was increased in the IHT group, without changes in blood LA concentration. These findings suggest that the increase in mRNA for MCT1 facilitated LA utilization, which caused a slower decline in pH during exercise [12]. In our study, the increase in glycolytic activity of muscle tissue was observed in the experimental group which performed the IHT training, what led to a significant increase in ΔLA concentration (28%) after the double Wingate test. This was correlated with significantly greater total work and mean power in both the first and the second Wingate tests. The improvement in glycolytic muscle tissue activity, and the significantly greater work led to the reduction in post-exercise ΔpH (33.5%), ΔBE (32.2%) and ΔHCO_3_act (16.1%). Similar changes were not found in the control group (C) that trained under normoxic conditions. On the other hand, these results are not consistent with the most recent study of Millet et al. [42] who examined the effectiveness of the IHT method in terms of improvement of anaerobic capacity. The study did not reveal any improvement in exercise performance. Furthermore, no changes were found in terms of MCT1 and MCT4 protein concentration in the skeletal muscle. The authors suggest that the lack of changes in this area was caused by insufficient exposure to hypoxia. With respect to previous reports, one should also take into consideration the type of training stimuli which was discussed above.

It is well established that regulation of pH depends on the concentration and activation of MCT1 and MCT4 proteins and on the activity of carbonic anhydrase (CA), which is both the recipient and donor of H^+^, by regulation of the rate of transport of H^+^ and HCO_3_^-^ [41,43]. Consistent with these reports Juel et al. [41] demonstrated an increase in CA (IV) in the muscle tissues following an eight-week exposure to hypobaric hypoxia (altitude conditions). Zoll et al. [12] also found an increase of mRNA for CA (IV) in the skeletal muscle after 6 weeks of an IHT protocol. The above reports represent strong evidence, showing that exposure to hypoxia modifies transport systems that participate in the buffering mechanisms of the human body.

Another often cited mechanism behind improvements in exercise performance induced by changes in acid-base homeostasis after exposure to hypoxia includes increased buffering capacity [44]. One of the forms of acute physiological response is hyperventilation that leads to the increase in vesicular PO_2_. This response leads to respiratory alkalosis, a reduction in PCO_2,_ and, consequently, to a reduction in concentration of H^+^, which causes an increase in pH [45]. This contributes to the release of bicarbonates from kidneys as they are one of the most basic buffers in the body [46]. The increasing LA and [H^+^] concentration that accompanies compensatory respiratory alkalosis during early stages of adaptation to altitude causes a greater decline in values of pH per mmol of the released ions H^+^ [47]. This mechanism may explain how altitude training contributes to improved buffering abilities of the muscle (βm) and inhibition of the degree of metabolic acidosis [48]. In line with this view is the study in which a high correlation between the relative increase in βm in the gastrocnemius muscle, and time of high-intensity running, was observed [49]. These results are also confirmed by the most recent study of Gore et al. [13], who found an improvement in βm by 18% in the vastus lateralis muscle after 23 nights spent at the altitude of 3,000m above sea level. However, these results are contradictory with the study of Clark et al. [40], who did not find such adaptive changes, which may be explained by less pronounced hypoxic stimuli compared to the findings of Gore et al. [13].

Another non-hematological adaptation mechanism which may contribute to the improvement in exercise performance during high-intensity short-term exercise is the increase in myoglobin concentration (Mb) in the muscle tissue. The major function of Mb is to store O_2_ in the striated muscle tissue. In such cases, Mb releases the stored particles of O_2_ and allows mitochondria to perform ATP synthesis through oxidative phosphorylation. Although exited data revealed the hypoxia impairs lipid metabolism driven from extra muscular sources, it cannot be excluded that by using intramuscular triglyceride as a supplementary substrate muscle can intensify oxidative metabolism [50]. Notably, it is well established that hypoxia significantly elevates plasma adrenaline levels [51].

The results of our study are not consistent with those conducted by Millet et al. [42], who examined the effectiveness of the IHT protocol in terms of glycolytic abilities. They examined cyclists who were involved in a three week interval training with two sets of three two-minute repetitions at maximal intensity obtained during a standard graded exercise test to exhaustion. The rest period between the repetitions equaled six minutes. According to our experience the lack of improvement in exercise capacity was attributable to insufficient load used in that study. In order to determine the load in the IHT training, which was performed at the altitude of 3,000m, the graded exercise test was repeated. The obtained maximal power was used to determine the intensity during the two-minute intervals. The participants from the control group performed the exercise with a mean load of 310W, whereas the load for the athletes from the experimental IHT group was adjusted to 260W. In comparison, the mean power for the upper limbs during the 30 second exercise was ~390W, both in the hypoxia and normoxia groups. This was possible because no limitations of exercise capacity are observed during the 30 second exercise, especially during work of upper limbs. A longer duration of exercise time (90 seconds) in the study by Mille et al. [42] caused a prolonged post-exercise oxygen debt and substantial contribution of aerobic metabolism, leading to a significant decline in generated power. Consequently, the training effect was oriented at improvement of aerobic rather than anaerobic capacity, which can be observed in the obtained results. Similar to the control groups, significant improvements in mean power generated during the two-minute exercise bouts were observed in the IHT group. However, the significantly lower oxygen uptake was found only in the hypoxia group, which suggests improvement in exercise energy expenditure.

It was observed that the exposure to high altitude (>3,000m above sea level) for a longer period of time (five to six weeks) leads to a 10–15% reduction in muscle volume in Himalayan climbers and a 20–25% reduction in the size of muscle tissues, without changes in distribution of individual fiber types [52,53,54]. A decline in oxidative muscle activity was demonstrated in mountaineers after return from the expeditions, whereas activity of glycolytic enzymes was increased [55]. This data indicates that the chronic exposure to high altitude through a reduction in overall exercise activity may have a harmful effect on muscle tissue as a result of decreased synthesis of muscle proteins [21,56], leading to atrophic changes in skeletal muscles. On the other hand, our latest reports [57] and the studies of other researchers [58,59] suggest that acute short-term exposure (one to two hours), especially if combined with resistance exercise has a contrary effect on muscle tissue by stimulation of muscle protein synthesis.

### Aerobic effects

In the present study, improved in relative values of VO_2max_ after IHT was associated with a changes in body mass. The lack of improvement in absolute values of VO_2max_ after IHT training observed in this study was most likely caused by insufficient time of a single repetition during the interval training protocol, which prevented a sufficient load to the cardiorespiratory system, and greater adaptive responses. The study with longer high-intensity IHT exercise bouts revealed the effectiveness of this method in improving VO_2max_ [3,11,12,60]. The lack of improvement in VO_2max_ observed in our study may be, at least partially, attributed to a predominate engagement of a rather small mass of upper body muscles that provide 70–85% of the propulsive forces during front crawl swimming [29]. Moreover, in relation to land-based exercise, swimming involves more technique-dependent breathing, with respiration being synchronized with swimming strokes [61], and the prone position implies venous return, which results in an increase in stroke volume (SV) and lowered HR during swimming [62]. Indeed, it has been reported that for the same level of oxygen uptake, heart rate (HR) and pulmonary ventilation during swimming were lower than those in land-based exercises [63,64]. Additionally, some data indicates a gap between the completion of the training protocol and the repetition of the incremental test as possible factor influencing the results of VO_2max_, because volume or/and intensity of applied training implies different recovery period [6].

Our previous studies [3] and those published by Dufour et al. [11] demonstrated a significant increase in aerobic capacity expressed by VO_2max_ by 4–5% and lactate threshold load by 4–8% after the IHT training under normobaric hypoxia conditions. The methodologies of these studies were similar, with participants involved in training based on lactate threshold intensity under hypoxic conditions (2,500m) from two to three times a week for a period of four to six weeks. Another four week experiment conducted on basketball players with the IHT method [1] revealed significant improvements in VO_2max_ by 6.5%. The improvements in VO_2max,_ were also documented by Roels et al. [60]. However, the training protocol applied in their study was different from the protocol used in the above mentioned studies [1,3,11]. That study used interval training with intensity at the VO_2max_ power level determined under normobaric hypoxia conditions. The IHT sessions were repeated twice a week (~115 min/week). Despite, the significant improvements in VO_2max_, the results of the experiment did not show increases in mean power generated during a 10-minute individual time trial, or in hematological indices after using the IHT methodology.

It should be emphasized that there are also other studies which have documented the non-positive effect of the IHT protocol on aerobic capacity in swimmers [65] and triathletes [66]. The lack of positive adaptive changes following the IHT in these studies was likely to be caused by insufficient exercise intensity during the IHT sessions. Furthermore, other examinations of IHT conducted in the hypobaric chamber in a group of triathletes [37] also did not reveal improvements in VO_2max_ levels. Exercise intensity during these examinations was adjusted individually, to 60–70% of heart rate reserve (HRR). Consequently, the participants performed the exercises only in the aerobic capacity zone. Such loads are used only to maintain exercise capacity and cannot contribute to improved performance.

The above reports suggest that training volume with high intensity plays a key role in eliciting significant adaptive changes in the body to IHT. An analysis of exercise protocols with IHT conducted by Bonetti and Hopkins [67] showed that exercise intensity is the key determinant of improved performance. Several researchers have indicated that IHT training with medium intensity (close to the anaerobic threshold) and large volume (20–30 min) led to improvements in aerobic capacity and exercise performance [1,3,11,12]. When high or maximal intensity was used in IHT protocols, with exercise duration under four minutes, no changes were found in aerobic capacity, but significant improvements were registered in anaerobic indices [60]. Furthermore, a lack of improvement was observed for prolonged low-intensity training sessions (below 80% VO_2max_) [28,60]. These results suggest that properly chosen exercise intensity is not the only factor that determines improvements in VO_2max_ following IHT protocols. The total training volume with properly adjusted exercise intensity is also essential to stimulate significant adaptive changes in well trained athletes.

The findings presented in this study are consistent with several previous reports [1,3,14,60,68] concerning the effect of intermittent hypoxic training (IHT) on hematological indices. Short-term cyclic (60min/three times a week) exposure to hypoxia combined with intensive physical exercise represents an insufficient stimuli to cause an increase in EPO blood levels and intensification of erythropoiesis. No significant changes in hematological indices were found in our study. Knaupp et al. [69] examined the relationships between the time of exposure to hypoxia and EPO blood serum concentration and found that exposure to hypoxia for 60 min did not cause a significant increase in EPO levels. A significant increase in this hormone (+50%) was observed after 120 min of exposure to hypoxia (F_I_O_2_ = 10.5%). Furthermore, Rusko et al. [70] found that the minimal time for improvement in hematological indices should be over 12 hours a day for at least three weeks at the altitude of 2,100–2,500m, which represents 250 hours of exposure to hypoxia.

Therefore, the significant increase in values of VO_2max_ and improvement in aerobic performance observed in previous studies after IHT are associated with non-hematological adaptive mechanisms due to hypoxia. The improvements in aerobic capacity and endurance performance are caused by muscular and systemic adaptations, which are either absent or found to a lesser degree after training under normoxic conditions [11,12]. Training under hypoxia may cause more dramatic changes in muscle tissues than following a conventional endurance training in normoxic conditions, which is related to increased skeletal muscle mitochondrial density, capillary-to-fiber ratio, and fiber cross sectional area, demonstrated in untrained individuals [21,56]. These adaptive changes are associated with an increase in hypoxia inducible factor-1α (HIF-1α), which is the global regulator of oxygen homeostasis and plays a critical role in the cardiovascular and respiratory responses to hypoxia [71].

## Conclusions

In conclusion, the most important finding of this study includes a significant improvement in anaerobic capacity and swimming performance after high-intensity IHT. However, this training protocol had no effect on absolute values of VO_2max_ and hematological variables. During the experiment, significant changes were observed in post-exercise acid-base equilibrium following the IHT protocol. The results of this study indicate that high-intensity intermittent hypoxic training (IHT) represents an effective training means for improving anaerobic capacity and swimming sprint performance.

Most scientists agree that these beneficial changes are caused by improved rate of phosphocreatine resynthesis, increased activity of phosphofructokinase (PFK-1) and glycolytic pathway enzymes, after training under hypoxic conditions. The above reports suggest that significant improvements in anaerobic capacity in IHT are possible through the application of maximal intensity without significant contribution of aerobic metabolism. Excitation of aerobic metabolism leads to a reduction in power generated during the IHT protocol compared to normoxia, which leads to a less pronounced response to training.

## Supporting information

S1 Fig Raw data from Fig 1A and 1B. H- experimental group, C–control group, S1—before training, S2 –after training, BM–body mass, Fat—fat content.
(PDF)

S2 Fig Raw data from Fig 2A and 2B. H- experimental group, C–control group S1—before training, S2 –after training,—100m—100m swim time trial, 200m—200m swim time trial.
(PDF)

S3 Fig Raw data from Fig 3A and 3B. H- experimental group, C–control group S1—before training, S2 –after training, UA rest—resting uric acid concentration, delta UA—changes in uric acid concentration after exercise.
(PDF)

S1 Table Raw data from Table 2. H- experimental group, C–control group, S1—before training, S2 –after training, RBC–red blood cell count, HGB- hemoglobin, HCT–hematocrit.
(PDF)

S2 Table Raw data from Table 3. H- experimental group, C–control group, S1—before training, S2 –after training, WR_max_—maximal workload during ramp test, VO_2max_—maximal oxygen uptake, RER_max_−maximal respiratory ratio during ramp test, VE_max_—maximal ventilation, HR_max−_maximal heart rate, ΔLA—increase in blood lactate concentration during ramp test, ΔLA12’res–decrease in blood lactate concentration after 12’ of recovery, ΔpH—blood pH changes, O_2_Sat- oxygen saturation at the end of the ramp test.
(PDF)

S3 Table Raw data from Table 4. H- experimental group, C–control group, S1—before training, S2 –after training, Ppeak–peak power, Pmean–mean power, ΔLA—increase in blood lactate concentration after double Wingate test, ΔLA12’res–decrease in blood lactate concentration after 12’ of recovery, ΔpH—blood pH changes after double Wingate test, O_2_Sat- oxygen saturation after two Wingate tests.
(PDF)
