# Supplementary material for: Intermittent hypoxic training improves anaerobic performance in competitive swimmers when implemented into a direct competition mesocycle
Source: PLoS One. 2017 Aug 1;12(8):e0180380. doi: 10.1371/journal.pone.0180380 (PMC5538675; doi:10.1371/journal.pone.0180380)
Supplement: S1 Fig — H- experimental group, C–control group, S1—before training, S2 –after training, BM–body mass, Fat—fat content. (PDF) [file pone.0180380.s001.pdf]

Group	Subject	BM S1 (kg)	BM S2 (kg)	Fat S1 (%)	Fat S2 (%)
H	1	71,4	69,4	5,3	5
H	2	74,6	74,6	7,5	7
H	3	78,3	79,1	11,6	10,9
H	4	71,5	72,2	8,7	7,3
H	5	74,3	71,7	10,2	8,9
H	6	88,5	88,5	16,7	16,7
H	7	77,9	77,8	7,6	7
H	8	75,4	76,7	6,9	5,3
C	1	70,1	70,1	11,2	11,1
C	2	69,8	69,9	7,6	7,2
C	3	77,3	77,6	11,3	10,4
C	4	82,6	81,8	11,3	10,2
C	5	78	78,8	9,3	9
C	6	64,1	64,5	8,2	7,4
C	7	77	76,9	8,2	7,9
