# Supplementary material for: Intermittent hypoxic training improves anaerobic performance in competitive swimmers when implemented into a direct competition mesocycle
Source: PLoS One. 2017 Aug 1;12(8):e0180380. doi: 10.1371/journal.pone.0180380 (PMC5538675; doi:10.1371/journal.pone.0180380)
Supplement: S2 Fig — H- experimental group, C–control group S1—before training, S2 –after training,—100m—100m swim time trial, 200m—200m swim time trial. (PDF) [file pone.0180380.s002.pdf]

Group	Subject	100m S1(s)	100m S2(s)	200m S1(s)	200m S2(s)
H	1	54,63	53,40	119,13	117,11
H	2	53,37	52,30	118,55	116,05
H	3	51,98	50,73	113,98	111,82
H	4	56,48	55,64	124,3	122,38
H	5	52,80	51,37	116,61	114,83
H	6	56,50	55,78	120,8	118,65
H	7	53,45	52,11	119,02	116,77
H	8	54,53	53,38	121,35	118,97
C	1	53,88	53,36	117,33	116,49
C	2	53,83	53,27	118,43	117,72
C	3	56,98	56,21	120,42	119,58
C	4	55,29	54,78	118,78	117,52
C	5	55,98	55,32	121,12	120,01
C	6	56,21	55,76	120,34	119,11
C	7	56,73	56,11	122,21	121,02
