# Supplementary material for: Intermittent hypoxic training improves anaerobic performance in competitive swimmers when implemented into a direct competition mesocycle
Source: PLoS One. 2017 Aug 1;12(8):e0180380. doi: 10.1371/journal.pone.0180380 (PMC5538675; doi:10.1371/journal.pone.0180380)
Supplement: S3 Fig — H- experimental group, C–control group S1—before training, S2 –after training, UA rest—resting uric acid concentration, delta UA—changes in uric acid concentration after exercise. (PDF) [file pone.0180380.s003.pdf]

Group	Subject	UArest S1	UArest S2	delta UA S1	delta UA S2
H	1	38,73	44,90	10,44	3,98
H	2	37,18	40,80	9,25	5,83
H	3	39,12	29,12	-1,48	4,26
H	4	38,04	31,40	14,18	8,45
H	5	23,35	30,43	9,40	4,63
H	6	29,92	36,71	11,25	4,78
H	7	32,91	26,88	5,36	2,62
H	8	39,30	38,65	11,27	3,82
C	1	42,18	36,28	6,69	3,65
C	2	36,46	29,96	6,42	3,39
C	3	48,90	44,85	2,19	2,13
C	4	56,30	53,98	1,56	4,23
C	5	44,43	39,99	5,35	3,85
C	6	35,80	28,23	4,15	3,99
C	7	44,55	43,63	3,12	3,29
