# Supplementary material for: Intermittent hypoxic training improves anaerobic performance in competitive swimmers when implemented into a direct competition mesocycle
Source: PLoS One. 2017 Aug 1;12(8):e0180380. doi: 10.1371/journal.pone.0180380 (PMC5538675; doi:10.1371/journal.pone.0180380)
Supplement: S1 Table — H- experimental group, C–control group, S1—before training, S2 –after training, RBC–red blood cell count, HGB- hemoglobin, HCT–hematocrit. (PDF) [file pone.0180380.s004.pdf]

Group	Subject	RBC S1 (mln/µl)	RBC S2 (mln/µl)	HGB S1 (g/dl)	HGB S2 (g/dl)	HCT S1 (%)	HCT S2 (%)
H	1	5,2	5,51	15,7	16,3	44,8	47,2
H	2	4,84	4,73	15,3	14,9	43,8	42,9
H	3	5,35	5,49	15,6	16,7	46,8	49,2
H	4	5,11	5,12	15,6	15,7	56,9	46,8
H	5	4,74	4,87	14,7	15,1	43,5	45,2
H	6	5	5,03	16,1	16,3	46,5	47,1
H	7	5,09	5,03	15	14,9	45,1	43,9
H	8	5,07	5,1	15,3	15,5	46,5	47,7
C	1	4,74	4,63	14,4	14,3	43,5	42,6
C	2	5,1	5,32	14,5	14,4	44,1	45,5
C	3	5,32	5,06	15,9	15,5	47,2	45,5
C	4	5,54	5,36	16,2	16,3	49,2	48,4
C	5	5,14	5,52	15,9	15,5	45,2	47,4
C	6	5,63	5,51	16,2	16,5	47,9	47,6
C	7	5,54	5,4	16,1	16	46,3	46,1
