# Supplementary material for: Intermittent hypoxic training improves anaerobic performance in competitive swimmers when implemented into a direct competition mesocycle
Source: PLoS One. 2017 Aug 1;12(8):e0180380. doi: 10.1371/journal.pone.0180380 (PMC5538675; doi:10.1371/journal.pone.0180380)
Supplement: S2 Table — H- experimental group, C–control group, S1—before training, S2 –after training, WRmax—maximal workload during ramp test, VO2max—maximal oxygen uptake, RERmax−maximal respiratory ratio during ramp test, VEmax—maximal ventilation, HRmax−maximal heart rate, ΔLA—increase in blood lactate concentration during ramp test, ΔLA12’res–decrease in blood lactate concentration after 12’ of recovery, ΔpH—blood pH changes, O2Sat- oxygen saturation at the end of the ramp test. (PDF) [file pone.0180380.s005.pdf]

Group	Subject	WRmax S1 (W)	Wrmax S2 (W)	VO2max S1 (l/min)	VO2max S2 (l/min)	VO2max S1 (ml/min/kg)	VO2max S2 (ml/kg/min)	RERmax S1	RERmax S2	VEmax S1 (l/min)	VEmax S2 (l/min)	HRmax S1 (bpm)	Hrmax S2 (bpm)	Delta LA S1 (mmol/l)	Delta LA S2 (mmol/l)
H	1	398	417	4,39	4,541	61	66	1,08	1,09	172,8	171	191	204	7,95	10,1
H	2	345	386	4,104	4,37	55	59	1,11	1,12	132,5	166	184	188	8,02	9,45
H	3	355	386	4,283	4,541	54	58	1,15	1,19	156,6	166	190	193	9,71	11,91
H	4	347	381	3,976	4,338	56	60	1,13	1,16	163,3	196,2	180	188	10,46	13,65
H	5	361	369	3,841	4,014	52	56	1,1	1,1	187	221,6	185	189	8,94	10,72
H	6	331	365	4,415	4,846	50	54	1,11	1,12	180	189,8	182	186	8,88	9,95
H	7	383	394	4,601	4,721	59	60	1,1	1,1	207,6	198,1	195	190	10,19	11,51
H	8	376	412	4,625	5,023	61	66	1,13	1,13	182	188,2	196	201	9,01	11,11
C	1	331	345	3,816	4,1	55	59	1,19	1,13	176,1	183	172	178	8,88	9,78
C	2	385	399	4,209	4,45	60	63	1,15	1,1	170	168,6	180	190	9,03	10,15
C	3	323	323	3,391	3,546	44	46	1,19	1,16	137,1	112,9	192	190	11,5	11,9
C	4	348	361	4,02	4,198	49	51	1,12	1,12	141,1	143,1	180	174	9,64	9,82
C	5	361	374	4,09	4,153	52	53	1,19	1,19	181,9	187,6	168	169	7,84	8,54
C	6	311	324	3,611	3,574	56	55	1,1	1,15	147	167,2	190	188	8,22	8,78
C	7	411	422	4,673	4,964	62	65	1,1	1,11	178	198,8	195	191	10,32	11,1

Group	Subject	Delta LAres12 S1 (mmol/l)	Delta LAres12 S2 (mmol/l)	Delta pH S1	Delta pH S2	SpO2 S1 (%)	SpO2 S2 (%)
H	1	1,72	3,02	-0,145	-0,175	94,9	94,6
H	2	1,91	2,58	-0,132	-0,133	94,5	94,1
H	3	3,39	2,75	-0,225	-0,261	96,7	95
H	4	2,89	1,93	-0,115	-0,158	96,1	93,3
H	5	3,14	4,52	-0,121	-0,172	97,2	96,1
H	6	4,08	5,16	-0,09	-0,108	96,4	97,1
H	7	2,31	2,56	-0,226	-0,214	95	93,1
H	8	3,87	3,45	-0,123	-0,207	94,8	94,3
C	1	3,62	3,15	-0,184	-0,191	94,5	96,6
C	2	2,46	2,38	-0,125	-0,155	94,8	94,2
C	3	2,41	5,47	-0,163	-0,161	94,1	95
C	4	1,45	2,22	-0,186	-0,175	95,1	92,6
C	5	2,87	3,02	-0,09	-0,089	96,5	94,6
C	6	2,24	2,09	-0,11	-0,121	96,4	96,6
C	7	2,18	2,55	-0,19	-0,185	95,8	94,8
