# Supplementary material for: Intermittent hypoxic training improves anaerobic performance in competitive swimmers when implemented into a direct competition mesocycle
Source: PLoS One. 2017 Aug 1;12(8):e0180380. doi: 10.1371/journal.pone.0180380 (PMC5538675; doi:10.1371/journal.pone.0180380)
Supplement: S3 Table — H- experimental group, C–control group, S1—before training, S2 –after training, Ppeak–peak power, Pmean–mean power, ΔLA—increase in blood lactate concentration after double Wingate test, ΔLA12’res–decrease in blood lactate concentration after 12’ of recovery, ΔpH—blood pH changes after double Wingate test, O2Sat- oxygen saturation after two Wingate tests. (PDF) [file pone.0180380.s006.pdf]

Group	Subject	Ppeak I S1 (W)	Ppeak I S2 (W)	Ppeak II S1 (W)	Ppeak II S2 (W)	Pmean I S1 (W)	Pmean I S2 (W)	Pmean II S1 (W)	Pmean II S2 (W)
H	1	567,62	704,3	559,25	687,25	403,3	468,98	388,24	452,76
H	2	865,03	897,5	610,05	731,55	440,1	503,87	419,54	452,87
H	3	759,77	777,2	703,65	768,46	473,2	508,01	455,76	494,68
H	4	755,3	779	665,09	798,57	444,3	462,46	423,44	441,85
H	5	598,19	661	587,45	676,42	433,1	492,41	398,86	492,29
H	6	902,57	938,6	714,64	1022	480,1	598,19	450,05	535,13
H	7	828,5	933,9	809,94	824,41	523	553,38	456,26	494,78
H	8	651,33	783,1	609,06	836,41	424,8	459,5	415,23	451,69
C	1	597,4	703,3	614,83	608,36	451,2	478,4	409,17	431,8
C	2	640,12	640,1	487,18	487,18	418,8	418,81	383,61	402,1
C	3	930,76	1050	841,22	815,8	456,3	496,26	398,5	412,5
C	4	852,73	863,4	622,1	951,61	407,1	431,27	405,7	422,56
C	5	952,68	1039	892,13	1064,7	453,1	464	425,6	437,61
C	6	690,2	913,2	609,23	814,64	366,8	405,41	349,94	365,2
C	7	671,79	1004	627,07	985,25	478,1	484,35	415,2	421,69

Group	Subject	Delta LA S1 (mmol/l)	Delta LA S2 (mmol/l)	Delta LA 12res S1 (mmol/l)	Delta LA 12res S2 (mmol/l)	Dela pH S1	Dela pH S2	SpO2 S1 (%)	SpO2 S2 (%)
H	1	6,93	11,33	1,2	1,28	-0,158	-0,211	97,1	96,1
H	2	9,11	11,51	3,13	2,81	-0,151	-0,184	96,6	96
H	3	8,17	9,64	1,61	1,29	-0,139	-0,18	97,1	96,3
H	4	10,86	9,57	2,81	2,26	-0,16	-0,176	96,8	95,9
H	5	5,9	14,85	2,24	2,33	-0,142	-0,299	97,1	96
H	6	8,65	13,92	2,85	2,25	-0,128	-0,226	97,4	96,5
H	7	13,79	13,97	1,8	1,24	-0,234	-0,314	96,6	96
H	8	9,93	10,22	1,97	2,29	-0,15	-0,197	96,8	96,4
C	1	11,68	11,34	3,24	2,15	-0,166	-0,226	96	97
C	2	13,18	13,18	2,35	2,35	-0,212	-0,212	97,1	97,1
C	3	17,46	14,05	1,57	2,15	-0,259	-0,252	94,8	95,5
C	4	13,52	12,55	1,77	1,24	-0,236	-0,248	95,2	96,7
C	5	10,29	8,85	3,35	3,13	-0,133	-0,142	96,2	95,9
C	6	12,46	12,13	2,26	2,97	-0,2	-0,193	95,8	96,5
C	7	10,12	10,51	0,55	1,07	-0,123	-0,134	87,2	87,2
